# Dicer ablation in osteoblasts by Runx2 driven cre-loxP recombination affects bone integrity, but not glucocorticoid-induced suppression of bone formation

**DOI:** 10.1038/srep32112

**Published:** 2016-08-24

**Authors:** Peng Liu, Mario Baumgart, Marco Groth, Jürgen Wittmann, Hans-Martin Jäck, Matthias Platzer, Jan P. Tuckermann, Ulrike Baschant

**Affiliations:** 1 Institute of Comparative Molecular Endocrinology (CME), Ulm University, Ulm, Germany; 2 Leibniz Institute on Aging– Fritz-Lipmann Institute, Jena, Germany; 3 Division of Molecular Immunology, Department of Internal Medicine III, Nikolaus-Fiebiger-Center, University of Erlangen-Nuremberg, Erlangen, Germany; 4 Department of Medicine III, Technische Universität Dresden, Germany

## Abstract

Glucocorticoid-induced osteoporosis (GIO) is one of the major side effects of long-term glucocorticoid (GC) therapy mediated mainly via the suppression of bone formation and osteoblast differentiation independently of GC receptor (GR) dimerization. Since microRNAs play a critical role in osteoblast differentiation processes, we investigated the role of Dicer dependent microRNAs in the GC-induced suppression of osteoblast differentiation. MicroRNA sequencing of dexamethasone-treated wild-type and GR dimer-deficient mesenchymal stromal cells revealed GC-controlled miRNA expression in a GR dimer-dependent and GR dimer-independent manner. To determine the functional relevance of mature miRNAs in GC-induced osteoblast suppression, mice with an osteoblast-specific deletion of Dicer (Dicer^Runx2Cre^) were exposed to glucocorticoids. *In vitro* generated Dicer-deficient osteoblasts were treated with dexamethasone and analyzed for proliferation, differentiation and mineralization capacity. *In vivo*, abrogation of Dicer-dependent miRNA biogenesis in osteoblasts led to growth retardation and impaired bone formation. However, subjecting these mice to GIO showed that bone formation was similar reduced in Dicer^Runx2Cre^ mice and littermate control mice upon GC treatment. In line, differentiation of Dicer deficient osteoblasts was suppressed to the same extent as wild type cells by GC treatment. Therefore, Dicer-dependent small RNA biogenesis in osteoblasts plays only a minor role in the pathogenesis of GC-induced inhibition of bone formation.

Glucocorticoids (GCs) are widely used to treat chronic inflammatory disorders such as rheumatoid arthritis. However, pharmacological doses of GCs cause glucocorticoid-induced osteoporosis (GIO) in up to 50% of patients receiving long-term GC-therapy^1^. GCs affect bone integrity by influencing the balance of bone formation and bone resorption systemically and locally resulting in decreased bone formation and consequently in reduced bone mass. Local effects of GCs on bone tissue involve direct effects on the bone forming osteoblasts derived from mesenchymal stromal cells. GCs diminish osteoblast function mainly by inducing apoptosis^2^,^3^, inhibiting pre-confluential proliferation^4^,^5^ and by suppressing differentiation^6^. GC act mainly via the glucocorticoid receptor (GR) altering gene expression by acting as a monomer and a dimeric ligand induced transcription factor in a cell type specific context. We could demonstrate that for most anti-inflammatory processes GR dimerization is essential to actively resolve inflammation^7^,^8^,^9^,^10^. Recently we could also demonstrate a dynamic reprogramming of GR monomer binding sites and GR dimer binding sites in the genome during GC exposure^11^. Strikingly, for inducing osteoporosis GR monomer function is sufficient^6^. Upon GC exposure, mice with a GR dimerization defective receptor exhibit inhibition of bone formation and impairment of osteoblast differentiation to a similar extent as wild type mice. In these GR^dim^ knock-in mice, a point mutation in the GR abrogates the dimerization interface in the DNA binding domain of the GR resulting in strongly reduced dimeric binding of the GR, and subsequent reduced GR dimer dependent transcription^11^,^12^. However, transcriptional regulation by the GR monomer is preserved in these mice^11^,^12^. As GIO was retained in GR^dim^ mice^6^, transcriptomes executed by GR monomers are decisive to decrease bone mass during GIO.

MicroRNAs are small non-coding, single-stranded RNA molecules that induce post-transcriptional gene silencing through base pairing with their target mRNAs. They are approximately ~22 nucleotides in length and they are processed from double-stranded hairpin precursors by two RNase III proteins, Drosha and Dicer (reviewed in ref. 13). Dicer is responsible for the final step in miRNA processing and germline deletion of *Dicer1* is embryonically lethal in mice underscoring the essential role of Dicer in physiological processes including embryonic development^14^. Emerging evidences indicate that numerous miRNAs are also involved in the regulation of bone homeostasis as inactivation of Dicer leads to bone defects and impaired mineralization during embryonic stage^15^. In line, several miRNAs have been identified with enhancing or inhibitory functions in osteoblast differentiation^16^,^17^,^18^,^19^. Recent studies also demonstrated a regulation of miRNAs in various GC-mediated cellular processes such as cell proliferation, cell differentiation and apoptosis^20^,^21^,^22^,^23^. Furthermore, a few miRNAs have been shown to be involved in GC-induced suppression of osteogenesis^24^,^25^,^26^.

Thus, miRNA regulation by GCs might be also decisive in glucocorticoid-induced inhibition of osteoblast differentiation and bone formation. Therefore, the aim of this study was to assess the role of miRNA biogenesis in the pathogenesis of GIO, in particular focusing on the suppression of osteoblast function and the consequent inhibition of bone formation.

## Results

### MicroRNA sequencing of GC treated mesenchymal stromal cells revealed GR dimerization and GR monomer dependent miRNAs

**Figure 1:**
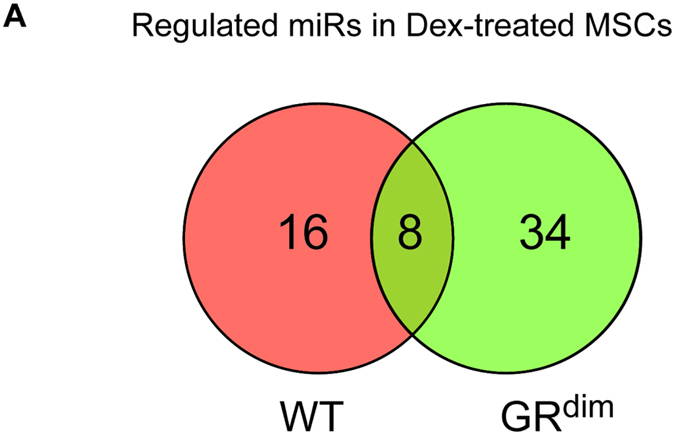
Small RNA sequencing of GC treated mesenchymal stromal cells revealed GR dimer and GR monomer dependent miRNAs. **(A)** Venn-Diagram showing GC-up-regulated and down-regulated miRNAs in wild type MSCs and GR^dim^ MSCs after Dex exposure in three (WT), respectively two (GR^dim^), independent biological replicates. Numbers indicate miRNAs highly regulated in WT MSCs (left), GR^dim^ MSCs (right) and common regulated miRNAs (middle), respectively.

We have previously shown that GCs mediate the suppression of bone formation in GIO mainly via the GR monomer in osteoblasts^6^. In order to determine miRNAs expression dependent on GR monomer function during osteoblast differentiation, we first aimed to identify identical expressed miRNAs in GC treated wildtype and GR dimer deficient osteoblasts by miRNA sequencing. We therefore cultured mesenchymal stromal cells derived from wild type and GR dimer deficient GR^dim^ mice under osteogenic conditions for 6 h with or without Dexamethasone, constructed a miRNA library and sequenced it using the Illumina technology. Venn analyses revealed sixteen differentially expressed miRNAs in wildtype primary mesenchymal cells that were treated with dexamethasone (Fig. 1A), of which eleven were up regulated (let-7 family, miR-125b, miR-146a, miR-148a + b, miR-152, miR-423) (Table S1) and five were down regulated (miR-1724a, miR-23a+b, miR-24-1,-2, miR-29a) (Table S1). In GR^dim^ derived primary osteoblasts we found 34 miRNAs differentially expressed upon Dex-treatment, of which 24 were up regulated and 10 down regulated (Fig. 1A + Table S1). In the total pool of differentially expressed miRNAs in Dex-treated wild type and GR^dim^ mesenchymal cells we found eight identical miRNAs that were up regulated (let-7a, let-7c, let-7f, let-7g, let-7i, miR-148a, miR-148b and miR-152) indicating GR monomer dependent GC regulation of miRNA expression (Table S1).

Thus, we observed miRNA regulation by GCs in mesenchymal stromal cells in a GR dimer and GR dimer independent manner.

### Ablation of dicer in osteoblasts causes growth retardation, low bone density and impairment of bone formation during postnatal development

**Figure 2:**
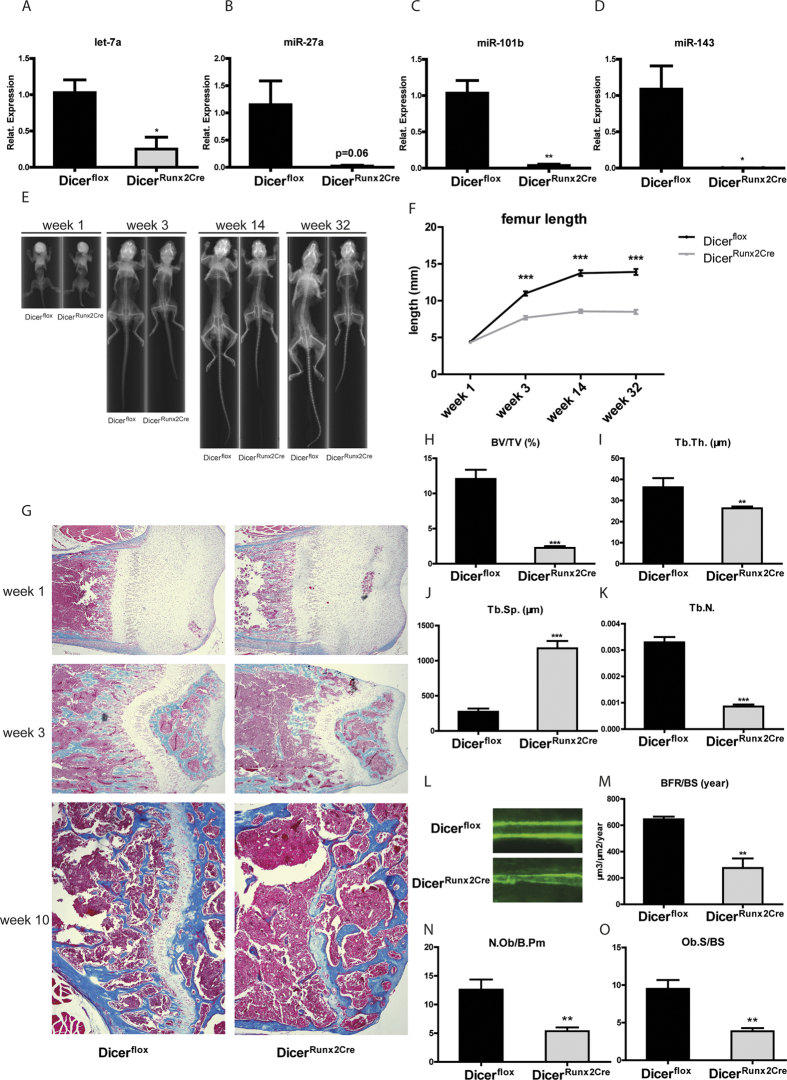
Ablation of dicer in osteoblast lineage causes growth retardation, low bone volume and impaired bone formation rate. (**A–D**) miRNA expression analysis of let-7a, miR-27a, miR-101b and miR-143 in femora of E15.5 embryo of *Dicer*^flox^ and *Dicer*^Runx2cre^ mice revealed abrogated miRNA biogenesis in bones of Dicer^Runx2Cre^ mice (n = 3). (**E**,**F**) Skeletal X-ray radiography revealed growth retardation (**E**) and reduced femur length (**F**) in *Dicer*^Runx2cre^ mice at indicated time points (week 1, week 3, week 14 and week 32). (**G**) Histological differences of femora of Dicer^flox^ and Dicer^Runx2Cre^ mice were visualized by trichrome staining of femora at indicated ages (week 1, week 3 and week 10). (**H–K**) Cancellous parameters such as bone volume per tissue volume (BV/TV), trabecular thickness (Tb.Th.), trabecular numbers (Tb.N.) and trabecular separation (Tb.Sp.) were measured in distal femora of 10-week-old *Dicer*^flox^ and *Dicer*^Runx2Cre^ mice by micro CT (n = 5). (**L**,**M)** Representative fluorescent micrographs of dual calcein labeling (**L**) and quantitative analysis (**M**) of bone formation rate per bone surface (BFR/BS) in femoral sections of 10-week-old *Dicer*^flox^ and *Dicer*^Runx2Cre^ mice are shown (n = 5). (**N**,**O**) Histomorphometry analysis of osteoblast number/bone perimeter (N. Ob/B.Pm) (**N**) and osteoblast surface/bone surface (Ob.S/B.S) (O) in trabecular bone on femoral sections of 10-week-old *Dicer*^flox^ and *Dicer*^Runx2Cre^ mice. *p < 0.05, **p < 0.01, ***p < 0.001.

To clarify to which extent miRNA regulation by GCs affects inhibition of bone formation we generated osteoblast-specific Dicer knockout mice. Therefore Dicer floxed mice were crossed with transgenic mice expressing Cre recombinase under the regulation of the Runx2 promoter. As confirmed by genomic PCR the Dicer encoding gene is almost completely ablated (Fig. S1A). We further confirmed abrogated miRNA biogenesis in Dicer^Runx2Cre^ mice by real-time PCR analysis of selected bone-related miRNAs in femurs of E15.5 embryos. MiRNA let-7a, miR-27a, miR-101b as well as miR-143 were all down regulated (Fig. 2A–D). Thus, miRNA expression is severely attenuated in Dicer^Runx2Cre^ mice.

Conditional deletion of Dicer alleles in pre-osteoblasts and osteoblasts results in significant growth retardation starting three weeks postnatally indicated by significant reduced torsal length as well as reduced body weight in Dicer^Runx2Cre^ mice compared to littermate control mice (Fig. S1B and S1C). Skeletal radiography also revealed shorter femur length in Dicer^Runx2Cre^ mice starting 3 weeks after birth. (Figure 2E,F). Furthermore trichrome staining for the distal part of femur depicted closure of the growth plate in Dicer^Runx2Cre^ mice at the age of 10 weeks (Fig. 2G).

Micro-computed tomography (Micro-CT) analysis of 10-week-old male mice revealed a highly significant decrease of bone density in Dicer^Runx2Cre^ mice compared to Dicer^flox^ mice (Fig. 2H). Accordingly, trabecular thickness (Tb. Th.) and trabecular numbers (Tb. N.) were decreased in Dicer^Runx2Cre^ mice whereas trabecular separation (Tb. Sp.) was increased (Fig. 2I–K).

Further dynamic histomorphometry analysis following dual calcein labeling in femurs of Dicer^flox^ and Dicer^Runx2Cre^ mice revealed a significantly reduced bone formation rate in Dicer^Runx2Cre^ mice compared to littermate control mice (Fig. 2L,M). In line, numbers of osteoblasts and osteoblast surface were significantly lower in Dicer^Runx2Cre^ mice (Fig. 2N,O). Numbers of osteocytes was slightly but not significantly changed in the femora of Dicer^Runx2Cre^ mice (Fig. S1D).

Osteoclast parameters and osteoblast-osteoclast crosstalk were not influenced by Dicer ablation in Runx2-expressing osteoblasts (Fig. S1E-S1G). To explore whether Dicer ablation in osteoblast influences osteoclasts, we cultured primary osteoblasts derived from calvaria of Dicer^flox^ mice that were crossed to mice expressing ubiquitously cre-ERT2 fusion (Dicer^gtRosa26CreERT2^ mice), allowing an inducible deletion of Dicer upon tamoxifen treatment. 4-hydroxytamoxifen (4-OHT) treatment completely ablated Dicer encoding gene (Fig. S2A) and Dicer protein (Fig. S2B) in primary osteoblasts.

Thus, absence of Dicer dependent miRNA biogenesis in Runx2 expressing, early osteoprogenitor cells impairs postnatal bone growth and results in decreased bone mass.

### Ablation of Dicer in osteoblasts does not affect dexamethasone inhibited proliferation and differentiation *in vitro*

**Figure 3:**
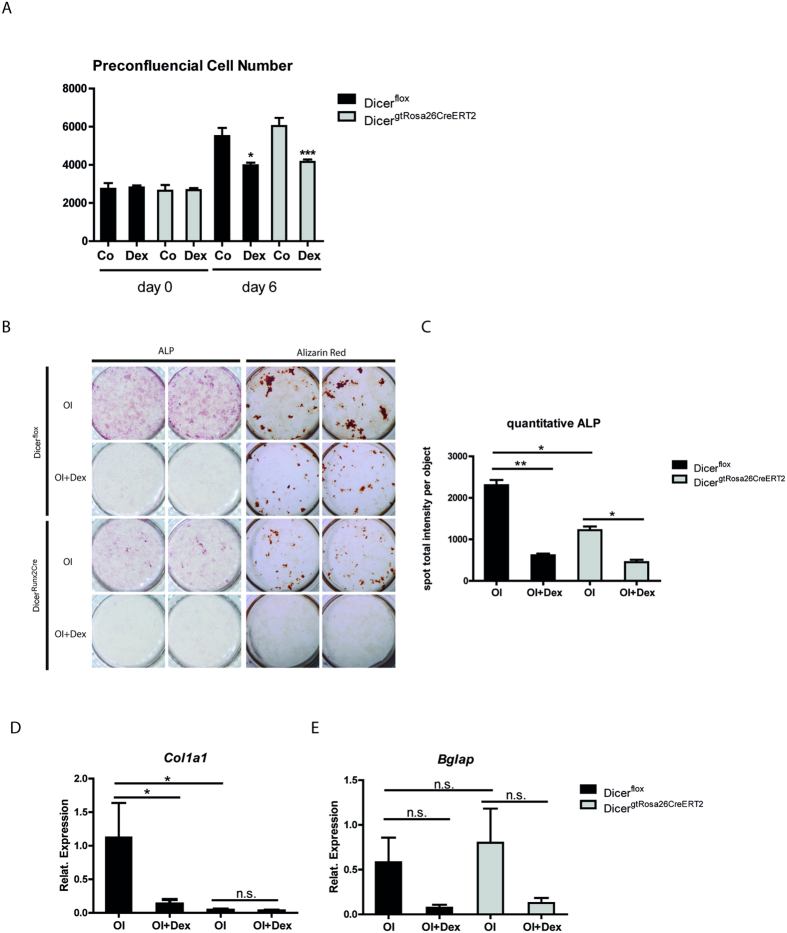
Dicer disruption does not affect dexamethasone inhibited proliferation and differentiation *in vitro*. (**A**) Primary osteoblasts were isolated from the calvaria of Dicer^gtRosa26CreERT2^ mice. Deletion of Dicer was induced by treating the cells with 1 μM 4-hydroxytamoxifen (4-OHT) for 3 consecutive days. After deletion, cells were treated with or without 10^−6^ M Dexamethasone (Dex) for 6 days. Cell numbers were assessed by automated microscope. (**B**) Osteoblast differentiation and mineralization of calvaria-derived primary osteoblasts of Dicer^gtRosa26CreERT2^ mice was assessed by alkaline phosphatase (day 10) and alizarin red (day 20) staining after Dicer deletion with 4-OHT and differentiation for 10 and 20 days, respectively. (**C**) Quantification of ALP activity was done by automated microscope. (**D**,**E**) Quantitative mRNA expression analysis of *Col1a1* (**D**) and *Bglap* (**E**) measured by qRT-PCR in calvaria-derived primary cells differentiated for 10 days or 20 days, respectively (n = 3). *p < 0.05, **p < 0.01, ***p < 0.001, n.s. not significant, OI vs OI + Dex.

Next, we investigated whether the absence of Dicer dependent mature miRNAs affects osteoblast proliferation and differentiation under GC treatment *in vitro*. Proliferation of preconfluent preosteoblasts was similar in both cultures of Dicer^flox^ and Dicer^gtRosa26CreERT2^ cells after 6 days (Fig. 3A). Treatment with dexamethasone strongly prevented expansion of wild type and Dicer^gtRosa26CreERT2^ cells (Fig. 3A). In contrast to proliferation of preconfluent osteoblasts, osteoblast differentiation reflected by alkaline phosphatase staining at day 10 of osteogenic induction and mineralization reflected by Alizarin Red staining at day 20 was decreased in cells derived from Dicer^gtRosa26ERT2^ mice (Fig. 3B, 3C). ALP activity and matrix mineralization were inhibited by Dex treatment in both, control and Dicer ablated cells (Fig. 3B,C). In line, the mRNA expression of the osteoblastic markers *Col1a1* and *Bglap* (Fig. 3D,E) tended to be reduced upon Dex treatment in Dicer deficient and wild type cells.

**Figure 4 f4:**
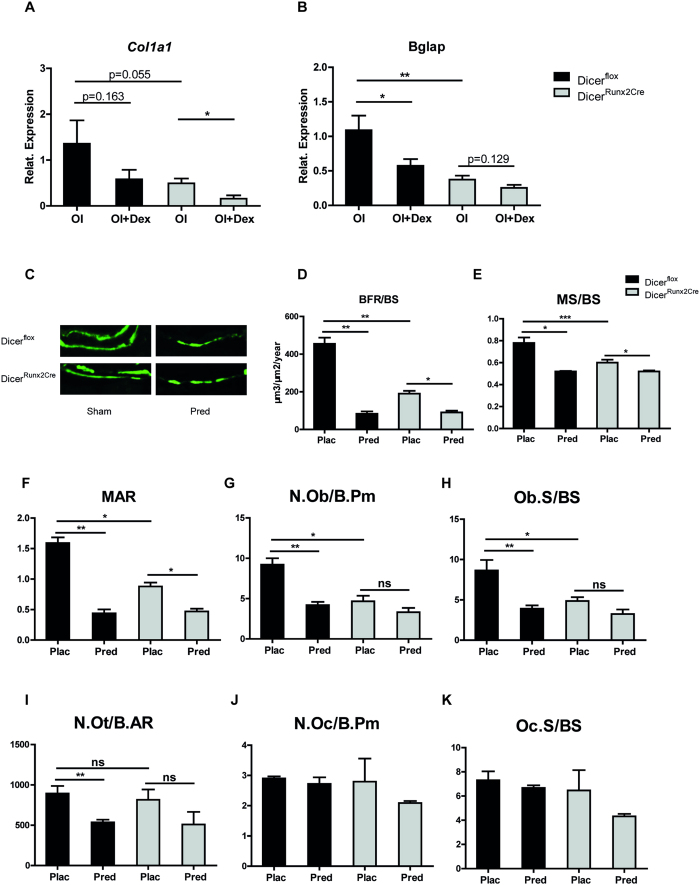
Ablation of dicer in Runx2 expressing osteoblasts does not prevent glucocorticoid inhibited bone formation. (**A**) E15.5 femora of Dicer^flox^ and Dicer^Runx2Cre^ mice were cultured for 3 days under osteogenic conditions with or without 10^−6^ M Dex. Gene expression levels of *Col1a1* (A) and *Osteocalcin* (**B**) were determined by qRT-PCR. (**C–E**) Fluorescent micrographs of dual calcein labeling (C) and quantitative analysis of bone formation rate per bone surface (BFR/BS) (D) as well as mineral apposition rate (MAR) (**E**) in femoral sections of Dicer^flox^ and Dicer^Runx2Cre^ mice under prednisolone treatment (Pred) or Control (Sham) are depicted. (**F–J**) Histomorphometry analysis of osteoblast number/bone perimeter (N. Ob/B.Pm) (F), osteoblast surface/bone surface (Ob.S/B.S) (**G**), osteocyte number/bone area (N. Ot/B.AR) (**H**), osteoclast number/bone perimeter (N. Oc/B.Pm) (**I**) and osteoclast surface/bone surface (Oc.S/B.S) (**J**) in femoral sections of Dicer^flox^ and Dicer^Runx2Cre^ mice under prednisolone treatment (Pred) or Control (Sham) is shown. *p < 0.05, **p < 0.01, ***p < 0.001, n.s. not significant, OI vs OI + Dex.

Next we cultured femora derived from E15.5 embryos under osteogenic conditions with or without Dexamethasone for 3 days. Gene expression analysis of osteoblastic markers revealed a significant downregulation of *Col1a1* and *Bglap2* in embryonic bones of Dicer^Runx2Cre^ mice compared to bones of Dicer^flox^ mice (Fig. 4A,B). Dexamethasone treatment reduced mRNA expression of *Col1a1* in femora of Dicer^flox^ and Dicer^Runx2Cre^ mice (Fig. 4A). *Bglap* expression was also suppressed by Dexamethasone in femora of Dicer^flox^ mice, but only by trend in Dexamethasone-treated bones of Dicer^Runx2Cre^ mice (Fig. 4B). Taken together, the absence of Dicer-dependent miRNA biogenesis does not overall affect the inhibitory effects of pharmacological doses of Dex on osteoblast differentiation markers in primary osteoblasts and organ culture.

### Glucocorticoids inhibit bone formation independent of dicer ablation in osteoblast

To define the role of Dicer dependent miRNAs in GC-induced suppression of bone formation *in vivo*, we treated Dicer^Runx2Cre^ mice with prednisolone for 14 days. Bone formation rate was similarly repressed in both Dicer^flox^ and Dicer^Runx2Cre^ mice upon prednisolone exposure (Fig. 4C,D). In line the mineral apposition rate was reduced in GC treated Dicer^Runx2Cre^ mice and in littermate control animals (Fig. 4F). In addition, prednisolone treatment reduced the mineralized surface in Dicer^flox^ and Dicer^Runx2Cre^ mice (Fig. 4E), indicating reduced numbers of osteoblasts on the femoral bone surface upon GC exposure.

Prednisolone-induced reduction of bone formation was accompanied by reduced numbers of osteoblasts, osteoblast surfaces and osteocytes in Dicer^flox^ mice as dynamic bone histomorphometry of femoral bone revealed (Fig. 4G–I). We also observed a non-significant trend of reduction of these parameters in Dicer^Runx2Cre^ mice (Fig. 4G–I) upon prednisolone treatment. Osteoclast parameters were similar in all four groups, independently of GC treatment, indicating that dicer ablation in osteoblasts may not change osteoblast-osteoclast cross-talk (Fig. 4J, [Fig f4]K). In summary, glucocorticoids inhibit bone formation and mineralization independent of dicer ablation in osteoblasts. Thus, our *in vitro* and *in vivo* data support the findings that Dicer dependent miRNA biogenesis only plays a minor role in the pathogenesis of GC-induced osteoporosis.

## Discussion

Pharmacological application of GCs in the treatment of chronic inflammation causes severe complications on the skeletal system leading to glucocorticoid-induced osteoporosis mainly due to GC-induced suppression of osteoblast function^27^.

MicroRNAs play a critical role in osteoblast differentiation processes. Even though there is evidence that miRNAs regulated by GCs influence osteoblast function^28^,^29^,^30^, the role of Dicer dependent microRNA biogenesis in GC-induced suppression of osteoblast differentiation has not been investigated *in vivo* yet. Here we show by *in vivo* analysis of pre-osteoblast specific deletion of Dicer that Dicer dependent miRNA biogenesis in early pre-osteoblasts is indispensable for normal bone growth, osteoblast differentiation and bone formation. We further revealed that Dicer ablation in pre-osteoblasts has only a minor impact on GC-induced suppression of bone formation.

The significant role of Dicer generated miRNAs in osteogenesis was also previously shown in different mouse models where Dicer was bone-specific deleted^17^,^18^,^31^. Whereas deletion of Dicer in osteoclasts^31^ decreased bone resorption and bone formation, deletion in the osteoblast lineage affected bone formation at different levels. Conditional deletion of the Dicer enzyme in osteoprogenitors by Col1a1Cre mice induced embryonic lethality and impaired osteoblast formation^17^. Similarly, Dicer deletion in more committed osteoprogenitor cells by OCN-Cre also prevented osteoblast differentiation and mineralization at early age. This phenotype was reversed at 2 to 4 month of age to a higher bone mass^17^. Dicer^OsxCre^ mice exhibited a mortality of 30% at 8 weeks and surprisingly, trabecular bone mass was not altered at 6 weeks but cortical thickness and texture was affected^18^. X-Ray radiography and micro-CT analysis of Dicer^flox^ mice crossed to Runx2Cre mice used in this study here (Dicer^Runx2Cre^ mice) revealed a significant retardation of skeletal growth and a severe reduction in bone mass. We did not observe lethality in Dicer^Runx2Cre^ mice until week 32. Since we detected a strong decreased expression level of several microRNAs and no impaired survival the Dicer^Runx2Cre^ mouse is an excellent model to study the effects of impaired microRNA generation starting from early-differentiated osteoblasts throughout the osteoblastic lineage,

Specifically, deletion of Dicer in Runx2 expressing cells does not exert any effects on osteoclastogenesis as our co-cultures with osteoblasts and osteoclast precursors revealed.

By analyzing regulation of miRNA expression in wild type cells and cells expressing a GR with impaired dimerization^11^, we found several miRNAs up-regulated including let-7, miR-146a, miR-148a, miR-148b and miR-152 by GC treatment, some of them exclusively in wildtype cells, and not in GR^dim^ cells. Among the GC-induced down-regulated miRNAs was miR-29a. miR-29a was shown to target Dkk-1, an inhibitor of the osteogenic Wnt signaling pathway, thereby reducing Dkk1 levels and enhancing Wnt signaling^32^,^33^. Along this line, Wang and colleagues suggested that miRNA-29a signaling protects against glucocorticoid-induced reduction of osteoblast differentiation and mineralization^20^,^28^. For several up and down regulated miRNAs intact GR dimerization is essential. However, since we previously demonstrated that GR monomer is sufficient for impaired osteoblast differentiation and bone formation by GCs^6^, we reasoned that miRNAs regulated by the GR monomer would be more likely to be involved in the deleterious effects of GCs. We identified eight miRNAs fulfilling this criterion that were up regulated in wild type and GR dimer deficient cells. Thus, we show here for the first time GR monomer dependent GC regulation of miRNA expression. Among the GR monomer dependent miRNAs were miR-148a and let-7i, whose expression was reported to be involved in differentiation of mesenchymal stromal cells towards osteoblasts^34^. Furthermore, we identified miR-152. Miao *et al*. showed that increased levels of miR-152 inhibited Wnt signaling, one of the most important pathways for maintaining bone homeostasis, by up regulating the negative Wnt regulator SFRP4^35^.

Despite these different molecular mechanisms of the GR to regulate miRNAs annotated for osteoblast function, we suggest that this regulation – at least of Dicer-dependent miRNAs - has a minor relevance for the deleterious effects on bone formation of GCs *in vivo*.

Our most striking finding was that osteoblast differentiation *ex vivo* and bone formation *in vivo* was similarly repressed by GC exposure in Dicer^flox^ and Dicer^Runx2Cre^ mice. The seemingly discrepancy to the function of GC-regulated miRNAs influencing osteoblasts revealed by^22^,^24^,^25^,^26^ could be explained that these studies focused on proliferation, which was impaired by anti-miRNA oligonucleotide against single miRNAs, but did not include differentiation^26^. Furthermore these results were mainly obtained by studying isolated mesenchymal stromal cells (derived from different tissues) or osteoblasts *ex vivo*.

However our study cannot exclude the functional involvement of miRNAs that mature independent of Dicer^36^ in the suppression of bone formation in GIO. Future analysis of *in vivo* loss and gain of functions studies of individual miRNAs will clarify the degree of Dicer independent miRNA effects in GIO. Thus, we cannot completely rule out that miRNAs play a role in glucocorticoid-induced changes of bone microarchitecture.

In conclusion, our results demonstrate that particular miRNAs are regulated by GCs in osteoblasts in a GR dimer and monomer dependent manner. Importantly, the abrogation of Dicer dependent miRNA regulation *in vivo* using Runx2Cre mice crossed to Dicer^flox^ mice reveal a model with viable mice with impaired bone growth and bone mass, underscoring the role of miRNAs for bone integrity. Our study further reveals that the regulation of Dicer generated miRNAs is dispensable during GC inhibition of bone formation. We conclude therefore that Dicer generated miRNAs are no suitable targets to prevent GC induced osteoporosis during long term GC therapy.

## Materials and Methods

### Declaration of approval for animal experiments

All experiments involving animals were approved by the Regierungspräsidium Tübingen, Germany.

### Mice and glucocorticoid-induced osteoporosis models

All mice were kept under standardized conditions with water and food *ad libitum* in specific pathogen-free animal facilities. Procedures for performing animal experiments were in accordance with the approved license from the Regierungspräsidium Tübingen, Germany. *Dicer*^Runx2Cre^ and *Dicer*^gtRosaCreERT2^ mice were generated by intercrossing *Dicer*^flox^ mice^37^ with Runx2Cre transgenic mice^38^ and gtRosaCreERT2 mice (Taconic artemis, Köln, Germany). Prednisolone was applied for 14 days by subcutaneous implantation of slow-release pellets, resulting in a dose of 12.5 mg/kg/d (15 mg; 60 day release; innovative research of America, Inc.) in 14-week-old male mice on a mixed background (C57BL/6, 129SV). GR^dim^ mice^39^ were backcrossed for at least 5 generations to FVB/N background.

### Histomorphometry

Static and dynamic histomorphometry was performed on undecalcified and decalcified femoral sections of mice receiving double calcein (Sigma-Aldrich, St. Louis, USA) i.p. injections, as described previously^40^,^41^ using Osteometrics system (Osteometrics, Decatur, USA).

### Micro-computed tomography (Micro CT)

Femora were analyzed using a SkyScan 1174 compact micro CT (Bruker, Billerica, USA) equipped with an X-ray tube working at 80 kV/100 μA. Resolution was 6.2 μm, rotation step was fixed at 0.40°, and a 0.5 mm aluminum filter was used. For reconstruction of femora, region of interest was defined 0.3 mm apart from the distal growth plate into the diaphysis spanning 1.8 mm. Trabecular bone volume/tissue volume (BV/TV), trabecular thickness (Tb.Th.), trabecular separation (Tb. Sp.) and trabecular number (Tb.N.) were determined according to guidelines by ASBMR Histomorphometry Nomenclature Committee^41^,^42^. X-ray radiograph was obtained by 70 μm resolution (La Theta, Aloka, Tokyo, Japan).

### Osteoblast differentiation and embryonic bone culture

Primary osteoblasts isolated from calvaria of neonatal *Dicer*^gtRosaCreERT2^ mice (postnatal day 3–5) by sequential digestions were cultivated as previously described^38^. The cells were exposed to 1 μM 4-hydroxytamoxifen (4-OHT) (Sigma-Aldrich, St. Louis, USA) for 3 days and subsequently subjected to osteogenic induction medium (Alpha-MEM containing 10% fetal bovine serum and 1% penicillin/streptomycin, supplemented with 100 mg/ml sodium ascorbate and 5 mM beta-glycerol phosphate, Sigma-Aldrich, St. Louis, USA) with or without 1 μM Dexamethasone (Sigma-Aldrich, St. Louis, USA). Osteoblast differentiation was determined by ALP staining (Sigma-Aldrich, St. Louis, USA) and Alizarin Red staining (Sigma-Aldrich, St. Louis, USA) at indicated time points. Quantitative ALP staining was performed using ELF phosphatase detection kit (ATCC, UK), and quantified by using cellomics arrayscan V^TI^ automated microscope (Thermo-Fisher Scientific, Waltham, USA).

Embryonic bones were isolated at day E15.5 from Dicer^Runx2Cre^ mice and cultured for three days in osteogenic induction medium (as described for the calvaria derived osteoblasts) with or without 1 μM Dexamethasone (Sigma-Aldrich, St. Louis, USA).

### miRNA sequencing

Total RNA was isolated (Trizol reagent, Invitrogen) from wild type and GR^dim^ derived mesenchymal stromal cells that were treated for 6h with osteogenic induction medium with or without 10^−6^ M Dexamethasone. Quality check and quantification was performed with Agilent Bioanalyzer 2100 and Agilent RNA 6000 nano kit (Agilent Technologies). 1 μg of total RNA was used for miRNA library preparation using the Illumina small RNA v1.5 sample kit. Library preparation was performed as described^43^. Sequencing was conducted with the Illumina sequencing platform^44^. Sequencing was done using a GAIIx (Illumina) in single-read, 36 cycle mode without multiplexing of libraries (sequencing one library per lane). The reads were extracted in FastQ format using tool GA-Pipeline v1.4 (supported by Illumina). Sequencing resulted in 10–12 mio reads per sample.

### Analysis of sequencing data

Individual sequence reads with base quality scores were produced by Illumina sequencing. The data were analyzed by the use of CLC-workbench (CLCbio, Arhus, Denmark). After eliminating reads with low quality and trimming the 3′ adaptor sequence, the remaining 18- to 33-nt reads were grouped into unique sequence clusters. To exclude potential sequencing errors, only sequences with at least 30 occurrences were included. Annotation of sequence clusters was performed, allowing 2 mismatches and up to 2 additional bases at each end, by using the *Mus musculus* reference from miRBase v16.0. Expression values were normalized to reads per million (RPM). Differentially-expressed miRNAs were identified using Kal’s Z-test for pairwise comparison. Venn-analyses were performed to find commonly regulated miRNAs between the three (WT), respectively two (GR^dim^), independent replications of experiments.

### Coculture experiments

Primary osteoblast isolated from Dicer^gtRosaCreERT2^ mice were seeded as 8,000 cells per well in 96-well plates and treated with 1μM 4-OHT for 3 days as described above. Bone marrow cells from wild-type mice were isolated and 200,000 cells per well were added on top of primary osteoblasts cultivated in alpha-MEM supplemented with 10 nM 1,25-dihydroxyvitamin D3 (Sigma-Aldrich, St. Louis, USA). Medium was changed every 3 days. After 9 days, cells were fixed and stained for TRAP activity.

### RNA isolation and qRT-PCR

Primary osteoblasts were lysed and total RNA was isolated (Qiagen, Hilden, Germany). RNA was reverse-transcribed by cDNA kit (Applied Biosystems, Carlsbad, USA) and real-time qPCR was performed as previously described^45^. Primer sequences are available upon request.

### Western blot

Primary osteoblasts were lysed in lysis buffer (Cell Signaling, Danvers, USA) containing proteinase inhibitor (Roche, Basel, Switzerland). Total protein amount was determined by BCA assay (Pierce, Waltham, USA) and western blot was performed with antibodies against Dicer (1:1000; #3363, Cell Signaling).

### Statistical analysis

Data are presented as mean ± standard errors (SE). Statistical evaluations of two group comparisons were performed using a two-sided Student’s t-test. One-way analysis of variance (ANOVA) was used for experiments with more than two groups.

## Additional Information

**How to cite this article**: Liu, P. *et al*. Dicer ablation in osteoblasts by Runx2 driven cre-loxP recombination affects bone integrity but not glucocorticoid-induced suppression of bone formation. *Sci. Rep*. **6**, 32112; doi: 10.1038/srep32112 (2016).

## Supplementary Material

Supplementary Information
