# Supplementary material for: Dicer ablation in osteoblasts by Runx2 driven cre-loxP recombination affects bone integrity, but not glucocorticoid-induced suppression of bone formation
Source: Sci Rep. 2016 Aug 24;6:32112. doi: 10.1038/srep32112 (PMC4995469; doi:10.1038/srep32112)
Supplement: Supplementary Information [file srep32112-s1.pdf]

SUPPLEMENTARY INFORMATION

Dicer ablation in osteoblasts by Runx2 driven cre-loxP recombination affects bone integrity, but not glucocorticoid-induced suppression of bone formation

Peng Liu[1,2], Mario Baumgart[2], Marco Groth[2], Jürgen Wittmann[3], Hans-Martin Jäck[3], Matthias Platzer[2], Jan P. Tuckermann[1,2,*] and Ulrike Baschant [1,2,4,*]

[1]Institute of Comparative Molecular Endocrinology (CME), Ulm University, Ulm, Germany; [2]Leibniz Institute on Aging– Fritz-Lipmann Institute, Jena, Germany; [3]Division of Molecular Immunology, Department of Internal Medicine III, Nikolaus-Fiebiger-Center, University of Erlangen-Nuremberg, Erlangen, Germany; [4]Department of Medicine III, Technische Universität Dresden, Germany

*authors contributed equally.

Table S1: Differentially expressed miRNAs upon Dexamethasone treatment in wt and GR^{dim} MSC

**wt vs. wt + Dex**		**GR^{dim} vs. GR^{dim} + Dex**		**wt + Dex and GR^{dim} + Dex**
**Up-regulated miRs**	**Down-regulated miRs**	**Up-regulated miRs**	**Down-regulated miRs**	**Common up-regulated miRs**
let-7a-1/ let-7a-2	miR-1274a	let-7a-1/ let-7a-2	miR-143	let-7a-1/ let-7a-2
let-7c-1/ let-7c-2	miR-23a	let-7b	miR-193b	let-7c-1/ let-7c-2
let-7f-1/ let-7f-2	miR-23b	let-7c-1/ let-7c-2	miR-210	let-7g
let-7g	miR-24-1/ miR-24-2	let-7d	miR-25	let-7i
let-7i	miR-29a	let-7e	miR-30a	miR-148a/ miR-148b
mirR-125b-2/ mir-125b-1		let-7f-1/ let-7f-2	miR-320	miR-152
miR-146a		let-7g	miR-326	
miR-148a/ miR-148b		let-7i	miR-378	
miR-152		miR-103-1	miR-484	
miR-423		miR-130a	miR-671	
		miR-142		
		miR-146b		
		miR-148a/ miR-148b		
		miR-152		
		miR-17		
		miR-196a-1/ miR-196a-2		
		miR-199a-1/ miR-199a-2		
		miR-20a		
		miR-26a-1/ miR-26a-2		
		miR-27b		
		miR-3068		
		miR-31		
		miR-872		

Dex, Dexamethasone; MSC, mesenchymal stromal cells; wt, wild-type;

Fig. S1

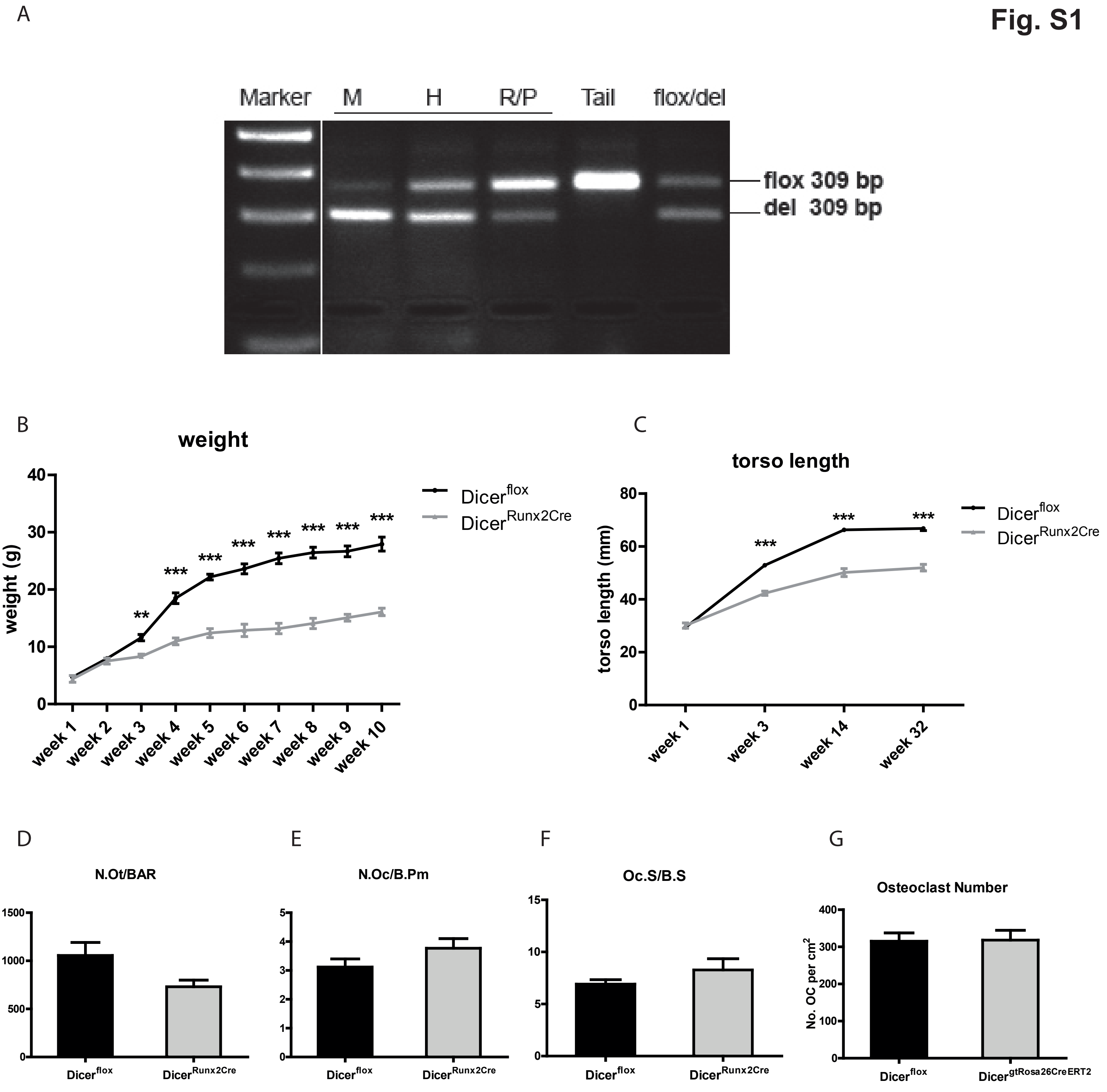
A
Marker
M
H
R/P
Tail
flox/del
flox 309 bp
del 309 bp
B
weight
weight (g)
0
10
20
30
40
Dicer^flox
Dicer^Runx2Cre
**
***
week 1
week 2
week 3
week 4
week 5
week 6
week 7
week 8
week 9
week 10
C
torso length
torso length (mm)
0
20
40
60
80
week 1
week 3
week 14
week 32
D
N.Ot/BAR
0
500
1000
1500
E
N.Oc/B.Pm
0
1
2
3
4
5
F
Oc.S/B.S
0
5
10
15
G
Osteoclast Number
No. OC per cm²
0
100
200
300
400
Dicer^flox
Dicer^Runx2Cre
Dicer^gtRosa26CreERT2

Fig.S2

A

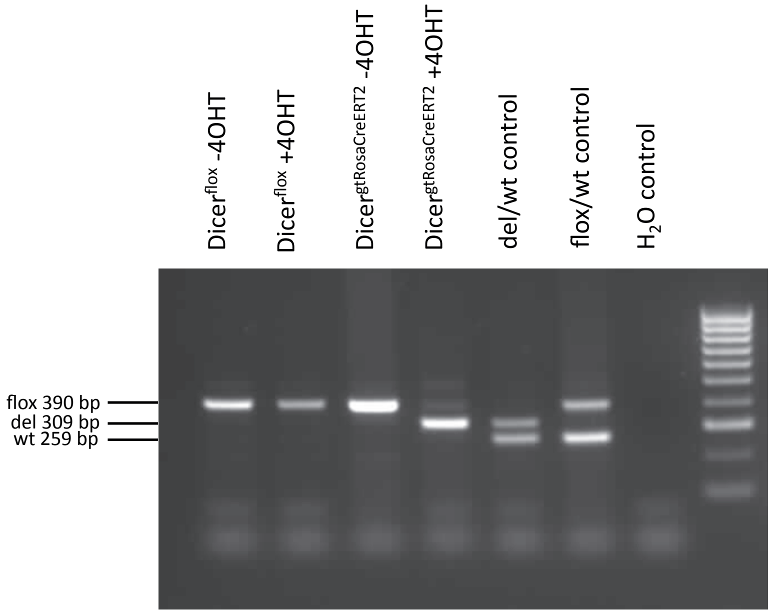

$Dicer^{flox}$ -4OHT
$Dicer^{flox}$ +4OHT
$Dicer^{gtRosaCreERT2}$ -4OHT
$Dicer^{gtRosaCreERT2}$ +4OHT
del/wt control
flox/wt control
H_2O control
flox 390 bp
del 309 bp
wt 259 bp

B

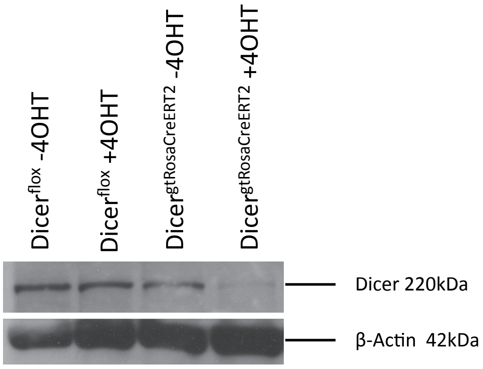

$Dicer^{flox}$ -4OHT
$Dicer^{flox}$ +4OHT
$Dicer^{gtRosaCreERT2}$ -4OHT
$Dicer^{gtRosaCreERT2}$ +4OHT
Dicer 220kDa
β-Actin 42kDa

SUPPLEMENTARY FIGURE LEGENDS

Fig. S1 Ablation of dicer in osteoblast lineage causes growth retardation, low bone density and the impairment of bone formation during postnatal development.

(**A**) Dicer deletion was demonstrated by genomic PCR of E15.5 femurs of Dicer^{flox} and Dicer^{Runx2Cre} mice.

(**B**) Male Dicer^{Runx2cre} and Dicer^{flox} mice were weighed postnatally from 1 week to 10 weeks.

(**C**) Torso length of *Dicer*^{flox} and *Dicer*^{Runx2cre} mice at indicated time point was measured by X-ray radiography.

(**D-F**) Histomorphometry of osteocyte number/bone area (N. Ot/BAR) (D), osteoclast number/bone perimeter (N. Oc/B.Pm) (E) and osteoclast surface/bone surface (Oc.S/B.S) **(F)** in trabecular bone on femoral sections of 10-week-old *Dicer*^{flox} and *Dicer*^{Runx2Cre} mice are depicted.

(**G**) Calvaria derived osteoblasts from *Dicer*^{flox} or *Dicer*^{gtRosaCreERT2} mice were co-cultured with wild type bone marrow cells with 10 nM 1,25-dihydroxyvitamin D3. After 9 days, number of multinucleated TRAP positive cells was determined (n=3).

** p<0.01, ***p<0.001.

Fig. S2 Inducible dicer disruption in primary osteoblast isolated from Dicer^{gtRosa26CreERT2} mice.

Primary osteoblasts isolated from the calvaria of Dicer^{flox} and Dicer^{gtRosa26CreERT2} mice were treated with 1 µM 4-OHT for 3 consecutive days. Dicer disruption was assessed by genomic PCR (A) and western blot (B).
